# Supplementary material for: Complex‐centric proteome profiling by SEC‐SWATH‐MS
Source: Mol Syst Biol. 2019 Jan 14;15(1):e8438. doi: 10.15252/msb.20188438 (PMC6346213; doi:10.15252/msb.20188438)
Supplement: Supplementary file 8 — Dataset EV7 [file MSB-15-e8438-s008.zip › feature_plots_string/P31930.pdf]

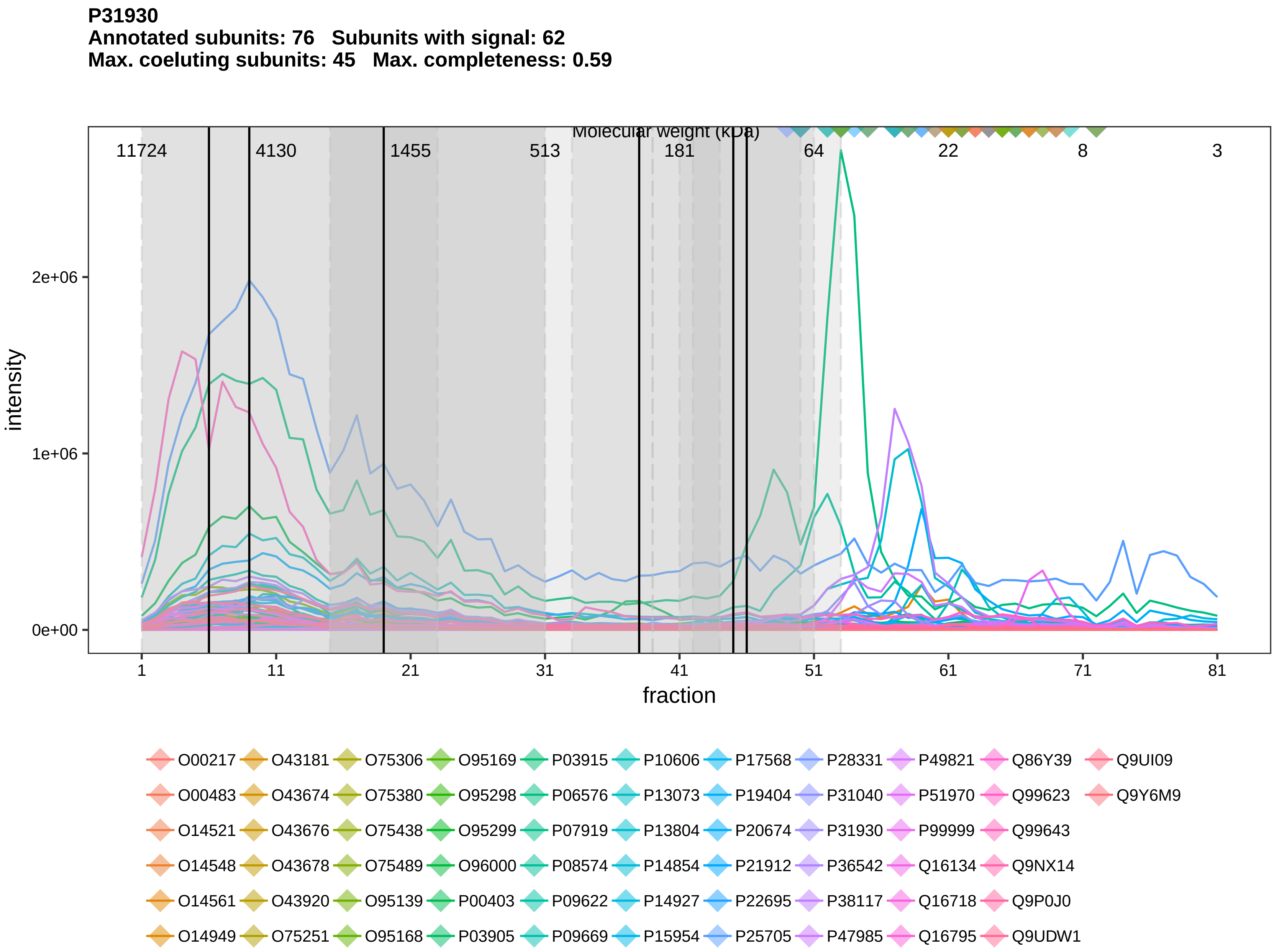
P31930
Annotated subunits: 76 Subunits with signal: 62
Max. coeluting subunits: 45 Max. completeness: 0.59
Molecular weight (kDa)
11724
4130
1455
513
181
64
22
8
3
intensity
2e+06
1e+06
0e+00
fraction
1
11
21
31
41
51
61
71
81
O00217 O43181 O75306 O95169 P03915 P10606 P17568 P28331 P49821 Q86Y39 Q9UI09
O00483 O43674 O75380 O95298 P06576 P13073 P19404 P31040 P51970 Q99623 Q9Y6M9
O14521 O43676 O75438 O95299 P07919 P13804 P20674 P31930 P99999 Q99643
O14548 O43678 O75489 O96000 P08574 P14854 P21912 P36542 Q16134 Q9NX14
O14561 O43920 O95139 P00403 P09622 P14927 P22695 P38117 Q16718 Q9P0J0
O14949 O75251 O95168 P03905 P09669 P15954 P25705 P47985 Q16795 Q9UDW1
